# Arabidopsis clathrin adaptor EPSIN1 but not MODIFIED TRANSPORT TO THE VACOULE1 contributes to effective plant immunity against pathogenic *Pseudomonas* bacteria

**DOI:** 10.1080/15592324.2022.2163337

**Published:** 2023-01-05

**Authors:** Kelly Mason, Erica LaMontagne-Mueller, Michael Sauer, Antje Heese

**Affiliations:** a University of Missouri-Columbia, Division of Biochemistry, Interdisciplinary Plant Group (IPG), Columbia, MO, USA; b Department of Plant Physiology, University of Potsdam, Potsdam, Germany

**Keywords:** Clathrin, plant immunity, EPS1, MTV1, ENTH, flg22, FLS2

## Abstract

In eukaryotes, EPSINs are Epsin *N*-terminal Homology (ENTH) domain-containing proteins that serve as monomeric clathrin adaptors at the plasma membrane (PM) or the *trans-*Golgi Network (TGN)/early endosomes (EE). The model plant *Arabidopsis thaliana* encodes for seven ENTH proteins, of which so far, only *At*EPSIN1 (*At*EPS1) and MODIFIED TRANSPORT TO THE VACUOLE1 (*At*MTV1) localize to the TGN/EE and contribute to cargo trafficking to both the cell surface and the vacuole. However, relatively little is known about role(s) of any plant EPSIN in governing physiological responses. We have recently shown that *At*EPS1 is a positive modulator of plant immune signaling and pattern-triggered immunity against flagellated *Pseudomonas syringae* pv. *tomato* (*Pto*) DC3000 bacteria. In *eps1* mutants, impaired immune responses correlate with reduced accumulation of the receptor FLAGELLIN SENSING2 (*At*FLS2) and the convergent immune co-receptor BRASSINOSTEROID INSENTIVE1-ASSOCIATED RECEPTOR KINASE1 (*At*BAK1) in the PM. Here, we report that in contrast to *At*EPS1, the TGN/EE-localized *At*MTV1 did not contribute significantly to immunity against pathogenic *Pto* DC3000 bacteria. We also compared the amino acid sequences, peptide motif structures and *in silico* tertiary structures of the ENTH domains of *At*EPS1 and *At*MTV1 in more detail. We conclude that despite sharing the classical tertiary alpha helical ENTH-domain structure and clathrin-binding motifs, the overall low amino acid identity and differences in peptide motifs may explain their role(s) in trafficking of some of the same as well as distinct cargo components to their site of function, with the latter potentially contributing to differences in physiological responses.

The *trans*-Golgi network (TGN) serves as an important sorting station to ensure that cargo proteins and other components reach their correct subcellular location for appropriate function. In plants, the TGN functionally overlaps with the early endosome (EE) and is thus referred to as the TGN/EE.^1–3^ As such, this cellular compartment is critical for post-Golgi trafficking of newly synthesized proteins to the plasma membrane (PM) or the vacuole; recycling of endocytosed proteins to the PM; and vacuolar trafficking of endocytosed PM proteins for degradation. Increasing evidence highlights the importance of clathrin-coated vesicle (CCV) components in cargo transport from the TGN/EE to their destination organelle. CCV formation occurs at both the PM and TGN/EE as a complex and highly coordinated process that involves distinct CCV core, adaptor, and accessory components.^3,4^ While the field is making inroads into identifying CCV components functioning at the TGN/EE and their cellular roles, their impact on physiological responses beyond plant growth and development remains largely elusive.^1–4^

We have recently advanced this limited understanding by identifying *Arabidopsis thaliana* EPSIN1 (*At*EPS1) as a positive modulator of plant immune responses and immunity against bacterial pathogens.^5^ *At*EPS1 is one of seven Epsin *N*-terminal Homology (ENTH)-domain proteins in Arabidopsis^6,7^ that are implicated as monomeric clathrin adaptors to link clathrin core components with other vesicular trafficking proteins and yet unknown lipid components to the site of CCV formation.^8,9^ Consistent with its localization to the TGN/EE,^8,9^ *At*EPS1 functions in trafficking of endogenous and ectopically expressed proteins to the PM or the vacuole.^5,9^ Notably, our work identifies *At*EPS1 as a critical component for the correct PM abundance of the plant immune receptor FLAGELLIN SENSING2 (*At*FLS2), the convergent co-receptor BRASSINOSTEROID INSENTIVE1-ASSOCIATED RECEP-TOR KINASE1 (*At*BAK1) and a small subset of structurally diverse PM proteins.^5^ We found that loss of *At*EPS1 does not affect ligand-induced endocytosis of *At*FLS2^5^ and constitutive bulk membrane endocytosis.^9^ Consistent with decreased PM accumulation of FLS2 and BAK1, *eps1* mutants display impaired immune responses to bacterial flg22, the active 22-amino acid derivative of bacterial flagellin, and other pathogen- or damage-associated molecular patterns^5^ that require PM-localized cognate host immune receptors and BAK1 for immune signaling. We also discovered that loss of *At*EPS1 results in increased susceptibility to flagellated *Pseudomonas syringae* pv. *tomato* (*Pto*) DC3000 bacteria, thus linking decreased PM accumulation of *At*FLS2 and *At*BAK1 to impaired immunity against bacterial leaf pathogens.^5^

So far, potential roles of the other six Arabidopsis ENTH-proteins in plant immunity have not been explored. Here, we focused on Arabidopsis MODIFIED TRANSPORT TO THE VACOULE1 (*At*MTV1) because so far, it is the only other Arabidopsis ENTH-domain containing protein that localizes to the TGN/EE and serves as a monomeric clathrin adaptor in cargo trafficking to the PM and the vacuole.^9,10^ To test whether similar to *At*EPS1,^5^ *At*MTV1 is required for effective resistance to virulent pathogenic *Pto* DC3000 bacteria, we infected leaves from 5-week-old plants Col-0 (wild-type), *eps1-2* (SAIL_394_G02) null mutant,^5^ and *mtv1-2* (Salk_061811) null mutant^10^ with the leaf bacterial pathogen *Pto* DC3000 (OD_600_ = 0.0005). Plant growth, plant infection by syringe-infiltration of *Pto* DC3000, leaf tissue processing and bacterial dilution plating were performed as described.^5^ No difference in bacterial growth was observed at 0-day post-infection (dpi) between either single mutant compared to Col-0 (Figure 1A; *p* > 0.05, n = 14), confirming that similar number of bacteria were initially infiltrated into leaves of each genotype. Consistent with our previous study,^5^ *eps1-2* null mutant plants showed enhanced susceptibility as seen by the increased number of *Pto* DC3000 compared to Col-0 at 3 dpi (Figure 1A; *p* = 0.0038, n = 33–34). In addition, *eps1-2* plants supported a significantly increased amount of *Pto* DC3000 bacterial colonization compared to *mtv1-2* plants (Figure 1A; *p* = 0.0416, n = 33–34). However, in contrast to *eps1-2* and Col-0, no statistically significant difference was observed between *mtv1-2* mutant and Col-0 plants at 3 dpi (Figure 1A; *p* > 0.05, n = 33–34). Similar results were obtained in each of the four independent experiments that were combined for Figure 1A, indicating that unlike *At*EPS1, *At*MTV1 does not play any significant role in defense against *Pto* DC3000 bacterial infection.

**Figure 1.**
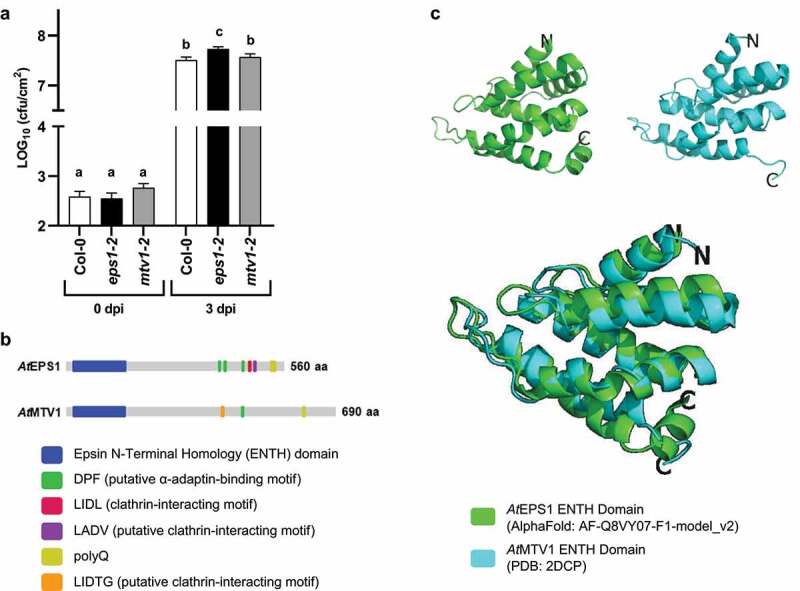
Loss of *At*EPS1 but not *At*MTV1 results in significant increased susceptibility to *Pto* DC3000 pathogenic bacteria. (a) Using syringe infiltration of *Pto* DC3000 (OD_600_ = 0.0005) in 5-week-old leaves, *eps1-2* but not *mtv1-2* null mutants displayed increased susceptibility at 3 days post-infiltration (dpi) compared to wild-type Col-0. cfu/cm^2^ = colony forming units per area. n=14 for 0 dpi and n=33-34 for 3 dpi with n being an individual leaf tissue sample from 8 and 16 different plants, respectively. Data are shown using LOG_10_ scale for y-axis. Values are mean ± SEM. Statistics were conducted using Student’s t test; same letter represents no significant difference; different letter represents a significant difference. Graph combines data from four independent experiments, with each individual experiment showing similar results. (b) Different domain and peptide motif architecture between *At*EPS1 and *At*MTV1 are indicative of distinct physiological functions. For individual motifs, letters indicate amino acids using their single letter codes. (c) Predicted structural fold of *At*EPS1 ENTH domain (green) is similar to that of resolved NMR structure for *At*MTV1 ENTH-domain (ref 14) (cyan).

The difference in function of *At*EPS1 but not *At*MTV1 in contributing significantly to resistance against *Pto*DC3000 is unlikely due to differences in their gene expression in Arabidopsis rosette leaves, the relevant tissue used for pathogen infection assays (Figure 1A) as *Pto* DC3000 is a leaf pathogen.^4,5^ Using Plant eFP Viewer public transcriptome repository (http://bar.utoronto.ca/eplant/)^11^ to visualize the gene expression ratio of *AtMTV1* (*AT3G16270*) to *AtEPS1* (*AT5G11710*) showed that both *ENTH* genes are expressed in rosette leaves with *AtMTV1* gene expression being only slightly decreased compared to *AtEPS1* with a Log2 ratio between −0.45 to 0 (Supplemental Figure S1). These are relatively minor expression differences compared to the high gene expression differences of *AtMTV1* to *AtEPS1* in other aerial tissues, namely mature pollen (Log2 ratio = 0.89), dry seed (Log2 ratio = 2.23) and late stages of embryo development (Log2 ratio = 0.89 to 2.23; see also ref 9) (Supplemental Figure S1). In fact, when we used ePLANT eFP viewer^11^ to visualize the relative gene expression levels of these two *ENTH* genes after syringe infiltration of rosette leaves with *Pto* DC3000 relative to its mock treatment at the corresponding hours post-infiltration, it was *AtMTV1* expression that was higher (Supplemental Figure S2A; Log2 ratio = 0.67) than *AtEPS1* (Supplemental Figure S2B; Log2 ratio = 0.15) at 2 hr-post-infiltration with this virulent pathogenic bacteria.

*At*EPS1 and *At*MTV1 are reported to function in trafficking of overlapping but also distinct cargo to the vacuole and the cell surface.^9^ Thus, it is tempting to speculate that *At*EPS1 but not *At*MTV1 may govern transport of proteins with immune function to their site of function. Because for eukaryotic EPSINs, differences in protein sequences, domain and peptide motif structure dictate their interaction with distinct vesicular trafficking proteins and cargo proteins,^6,7^ we performed a more detailed analysis comparing *At*EPS1 and *At*MTV1 proteins. Using EMBOSS Needle pairwise alignment (https://www.ebi.ac.uk/Tools/psa/emboss_needle/) that was visualized using JalView (https://www.jalview.org/) to compare full length protein sequences (Supplemental Figure S3), we observed that these two ENTH-proteins shared overall low amino acid sequence identity (17.7%) and similarities (29.6%) including within their ENTH-domains (Supplemental Figure S3). *At*EPS1 and *At*MTV1 also showed differences in their peptide motif architecture in the unstructured middle and C-terminal regions (Figure 1B; Supplemental Figure S3). The number of putative adaptor-binding peptide motifs differ, with *At*EPS1 possessing three but *At*MTV1 only one DPF peptide motif (Figure 1B; Supplemental Figure S3). *At*EPS1 and *At*MTV1 have distinct clathrin binding motifs in their unstructured C-terminus, with *At*MTV1 containing the putative clathrin-interacting peptide motif LIDTG (Figure 1B; Supplemental Figure S3) and *At*EPS1 containing the confirmed LIDL^8^ and the potential LADV clathrin-interacting motifs (Figure 1B, Supplemental Figure S3). Both *At*EPS1 and *At*MTV1 contain poly-glutamine (polyQ) stretches; but that of *At*EPS1 is longer consisting of eleven glutamines compared to the four glutamines in *At*MTV1. So far, roles of polyQs remain elusive for plant EPSINs; but polyQ stretches can facilitate protein-protein interactions and form cytoplasmic condensates in plants.^12^

Due to its low primary sequence similarity to other EPSIN paralogs (including *At*EPS1, Supplemental Figure S3), *At*MTV1 is annotated as a phylogenetically distant ENTH-protein.^6,9^ In some cases, its ENTH domain has been referred to as a VHS domain (www.uniprot.org; Q9C5H4), an N-terminal domain named after its occurrence in Vps27, Hrs and STAM proteins and that shares extremely similar α-helical folds with ENTH-domains.^13^ To assess whether the low primary sequence identity for their ENTH-domain may result in different tertiary structures, we downloaded the resolved Nuclear Magnetic Resonance (NMR) structure of *At*MTV1 ENTH domain as published encompassing amino acids 9–135^14^ from the Protein Data Bank (PDB) (2DCP). For comparison, we downloaded the predicted structure of *At*EPS1 ENTH domain (amino acids 27–152) (this study) from Alphafold (AF-Q8VY07-F1-model_v2), an open access protein structure prediction database.^15,16^ For molecular visualization, the two ENTH structures were aligned and analyzed using the open-source program PYMOL (https://pymol.org/2/). Notably, despite their relatively low amino acid sequence similarity, the ENTH-domains from *At*EPS1 and *At*MTV1 were predicted to share a compact globular structure composed of seven α-helical superhelix fold that were connected by loops of varying lengths (Figure 1C; Supplemental Movie 1). As such, our findings are consistent with reports for other eukaryotic ENTH-proteins.^6,13^

In conclusion, we provide evidence that *At*EPS1 but not *At*MTV1 plays an apparent role in effective immunity against *Pto* DC3000 (this study), thus demonstrating differences in the physiological roles of these two TGN/EE-localized Arabidopsis ENTH-proteins *in planta*. Diverse biological function is consistent with *At*EPS1 and *At*MTV1 a) showing low sequence identity and differences in peptide motif architectures (this study); b) localizing to different regions of the TGN/EE and preferentially bind to β-subunits of distinct AP complexes;^9^ and c) functioning in trafficking of overlapping as well as distinct cargo to the vacuole and the cell surface.^9^ In future studies, it will be interesting to delineate whether different peptide motifs and solvent-exposed amino acids in the ENTH-domains of *At*EPS1 or *At*MTV1 provide binding specificity for lipids and/or proteins necessary for trafficking of distinct cellular components to their subcellular site of function to confer physiological responses.

## Supplementary Material

Supplemental Material
