# Supplementary material for: Arabidopsis clathrin adaptor EPSIN1 but not MODIFIED TRANSPORT TO THE VACOULE1 contributes to effective plant immunity against pathogenic Pseudomonas bacteria
Source: Plant Signal Behav. 2023 Jan 5;18(1):2163337. doi: 10.1080/15592324.2022.2163337 (PMC9828777; doi:10.1080/15592324.2022.2163337)

Epsin N-Terminal Homology (ENTH) domain

DPF (putative α-adaptin-binding motif)

LIDL (clathrin-interacting motif)

LADV (putative clathrin-interacting motif)

LIDTG (putative clathrin-interacting motif)

polyQ

**Supplemental Figure S1. *At*EPS1 and *At*MTV1 share very low primary sequence similarity.**

Amino acid sequences were  gathered from Uniprot (EPS1:Q8VY07 and MTV1:Q9C5H4). Alignment was conducted using EMBOSS Needle pairwise alignment (<https://www.ebi.ac.uk/Tools/psa/emboss_needle/>) and visualized using JalView (<https://www.jalview.org/>) for full length proteins**.** Coloring of residues is based on conservation with a conservation visibility value of 30%. Darker blue shading represents shared identity, lighter blue shading represents similarity, and no shading represents no similarity. *At*EPS1 and *At*MTV1 have a sequence identity value of 17.7% and sequence similarity value of 29.6%. Calculations for sequence identity and similarity were done with EMBOSS Needle. Different color boxes identify specific domains and peptide motifs and were annotated based on Uniprot ([www.uniprot.org](http://www.uniprot.org/)) and ref 5.


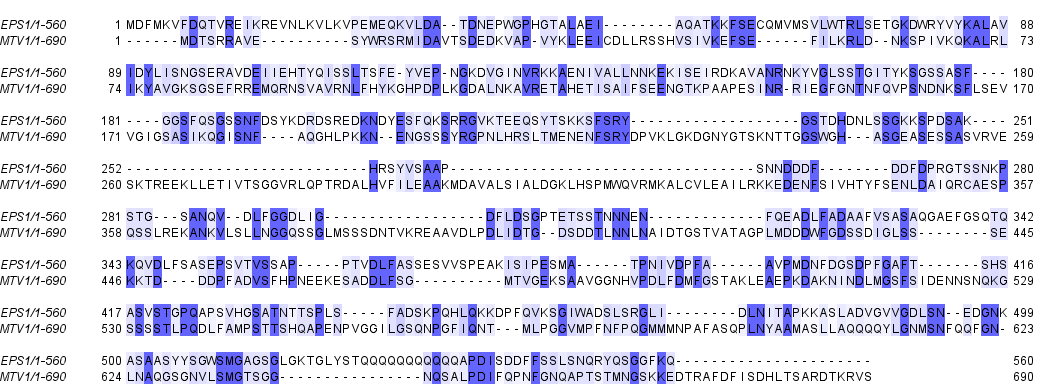

Supplement: Supplemental Material [file KPSB_A_2163337_SM6930.zip › Mason et al SupplementalFigure S1.docx]
