# Supplementary material for: Arabidopsis clathrin adaptor EPSIN1 but not MODIFIED TRANSPORT TO THE VACOULE1 contributes to effective plant immunity against pathogenic Pseudomonas bacteria
Source: Plant Signal Behav. 2023 Jan 5;18(1):2163337. doi: 10.1080/15592324.2022.2163337 (PMC9828777; doi:10.1080/15592324.2022.2163337)
Supplement: Supplemental Material [file KPSB_A_2163337_SM6930.zip › new Mason et al Suppl Figure S1 MTV1 to EPS1.docx]

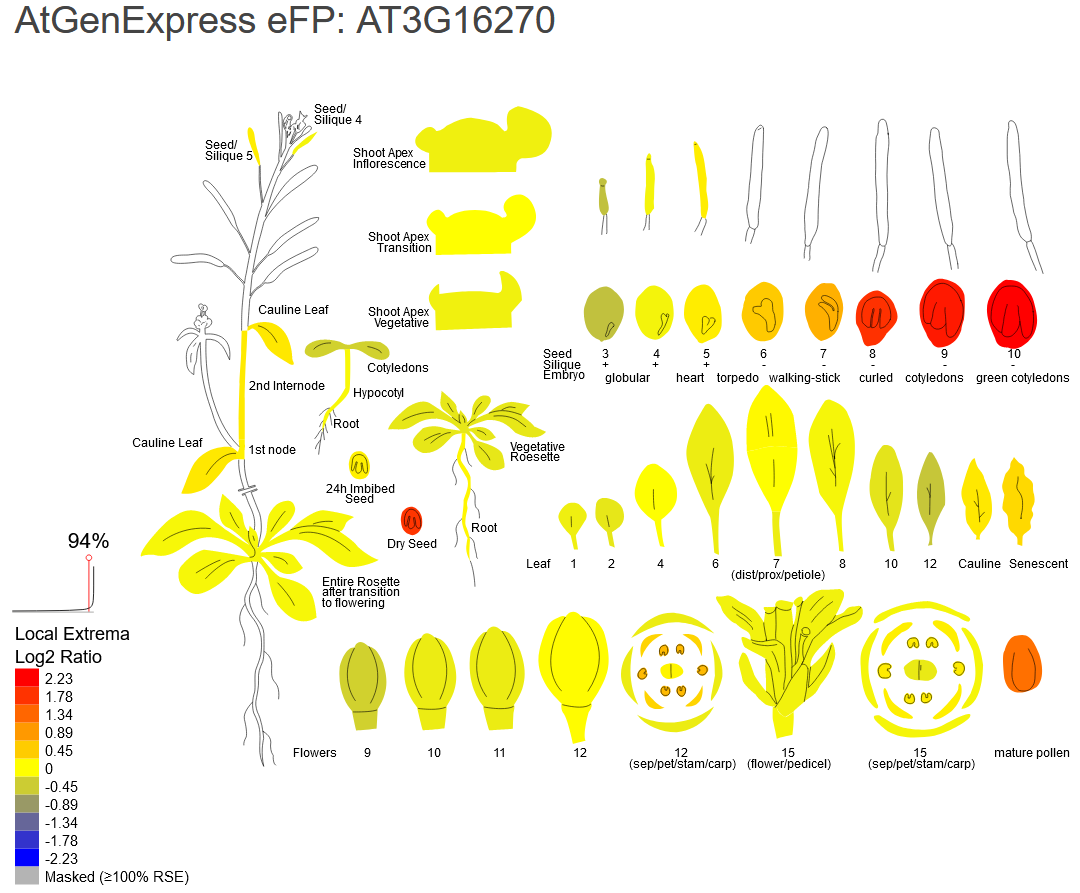

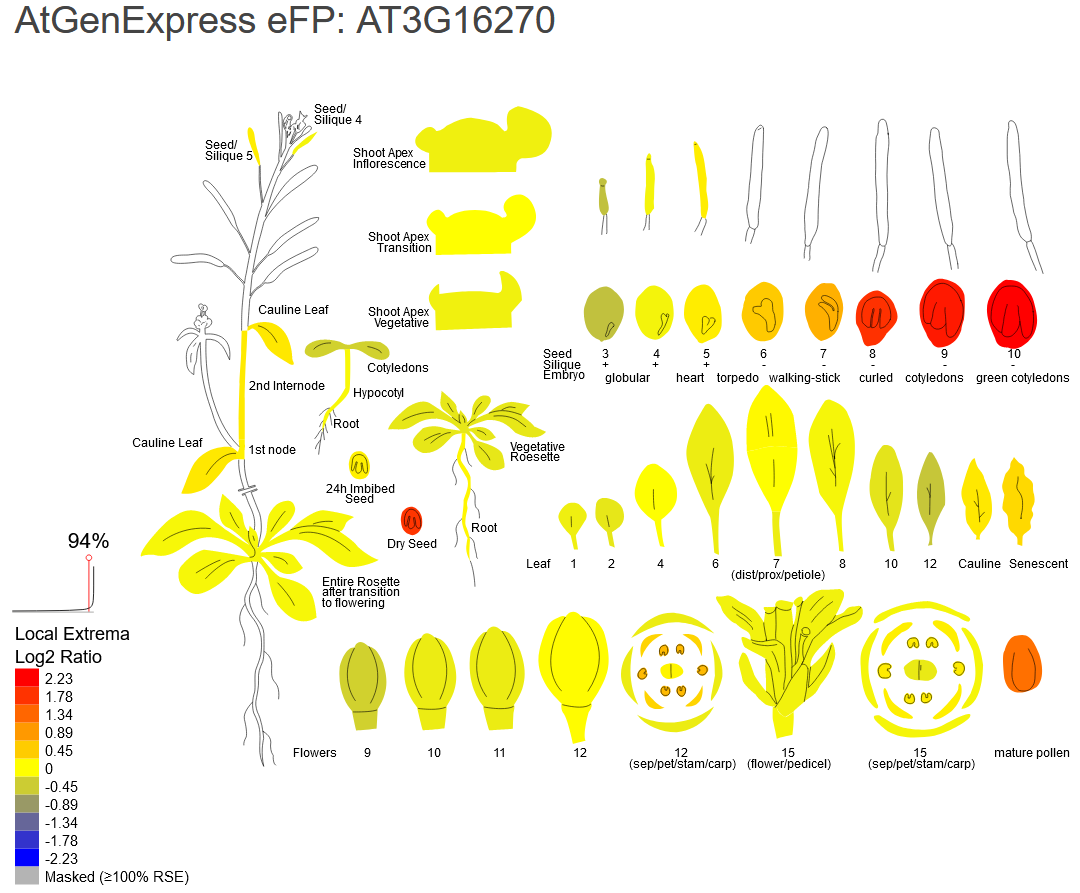


**Supplemental Figure S1. Minor gene expression differences between *AtMTV1* and *AtEPS1* in rosette leaves used for pathogen infection assays unlikely contribute to role of *AtEPS1* but not *AtMTV1* in plant immunity against *Pto* DC3000 bacteria.**

Ratio of *AtMTV1* (*AT3G16270*) to *AtEPS1* (*AT5G11710*) gene expression as visualized from the Plant eFP Viewer public transcriptome repository (<http://bar.utoronto.ca/eplant/>) (see ref 11). Gene expression data are depicted in relative mode (Log2 ratio) with values below 1 and blue color indicating higher EPSIN1 expression, and values above 1 and red color indicating higher MTV1 expression. Yellow, light green and light orange indicate no or relatively subtle differences in gene expression.
