# Supplementary material for: Arabidopsis clathrin adaptor EPSIN1 but not MODIFIED TRANSPORT TO THE VACOULE1 contributes to effective plant immunity against pathogenic Pseudomonas bacteria
Source: Plant Signal Behav. 2023 Jan 5;18(1):2163337. doi: 10.1080/15592324.2022.2163337 (PMC9828777; doi:10.1080/15592324.2022.2163337)
Supplement: Supplemental Material [file KPSB_A_2163337_SM6930.zip › new Mason et al Suppl Figure S2 ePlant PtoDC3000.docx]

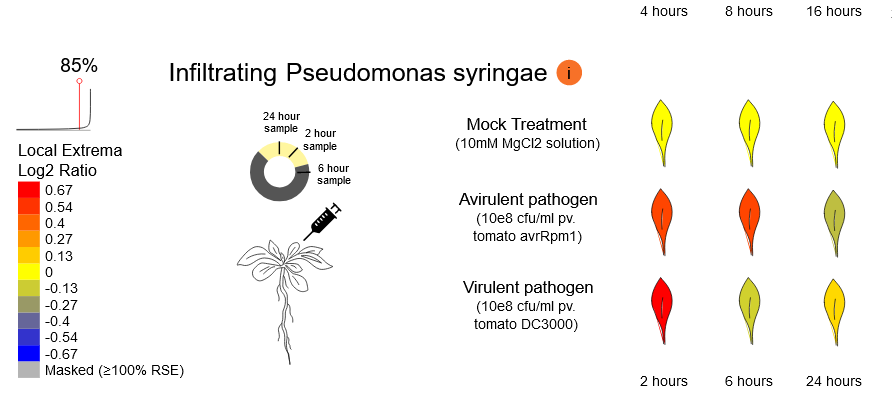


***AT3G16270 (AtMTV1*)**

**A**


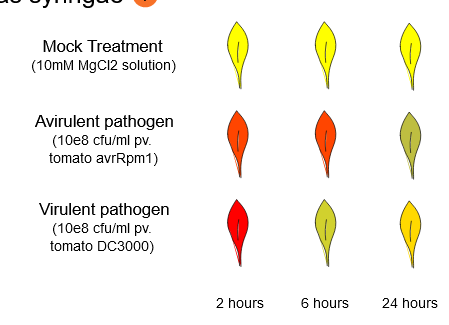

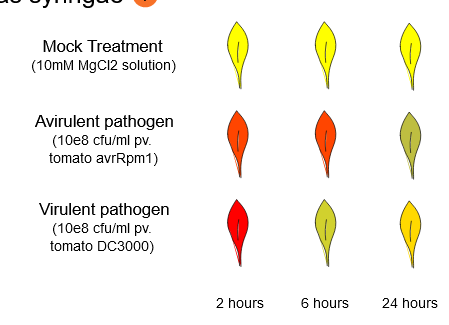


**0.67**

**0.1**

**- 0.12**

**B**

***AT5G11710 (AtEPS1)***

**A**


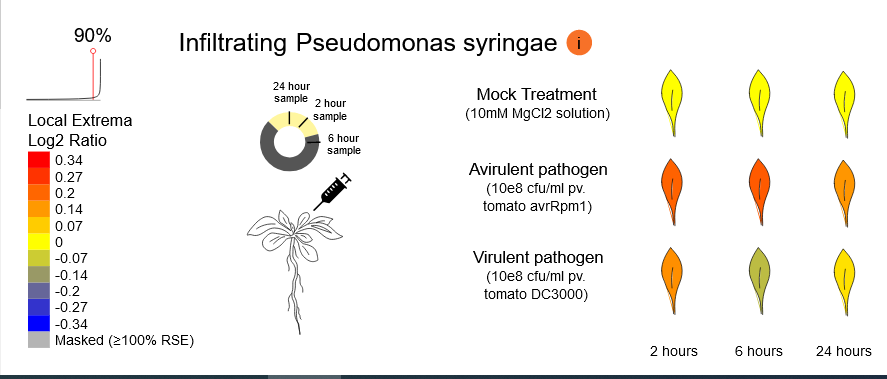


**B**


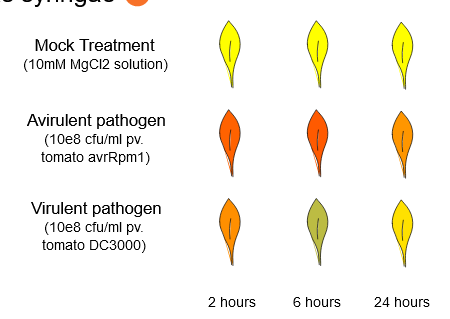

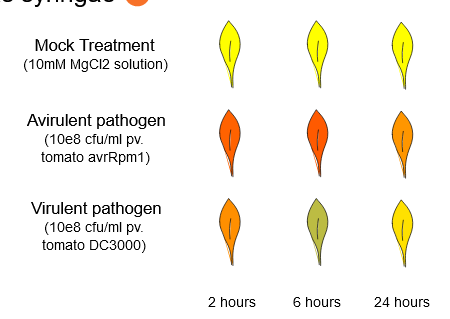


**0.15**

**0.04**

**- 0.09**

**Supplemental Figure S2. Relative gene expression of *AtMTV1* and *AtEPS1* in response to infiltration with virulent *Pto* DC3000 bacteria.**

Using ePLANT eFP viewer public transcriptome repository (see ref 11), relative gene expression levels are shown for *AtMTV1* **(A)** and *AtEPS1* **(B)** after syringe infiltration with virulent pathogenic *Pto* DC3000 (pv tomato DC3000) or corresponding Mock Treatment (10 mM MgCl_2_ solution) at 2-, 6-, and 24-hours post-infiltration. Numbers in bold above the leaf images are the numerical Log2 of fold change for gene expression levels after *Pto* DC3000 treatment relative to corresponding mock treatment. Values above 0.2 and red color indicate increased expression; values below - 0.2 and blue indicate decreased expression.
