# Supplementary material for: Arabidopsis clathrin adaptor EPSIN1 but not MODIFIED TRANSPORT TO THE VACOULE1 contributes to effective plant immunity against pathogenic Pseudomonas bacteria
Source: Plant Signal Behav. 2023 Jan 5;18(1):2163337. doi: 10.1080/15592324.2022.2163337 (PMC9828777; doi:10.1080/15592324.2022.2163337)
Supplement: Supplemental Material [file KPSB_A_2163337_SM6930.zip › same Mason et al SupplementalMovie1.pptx]

## Slide 1
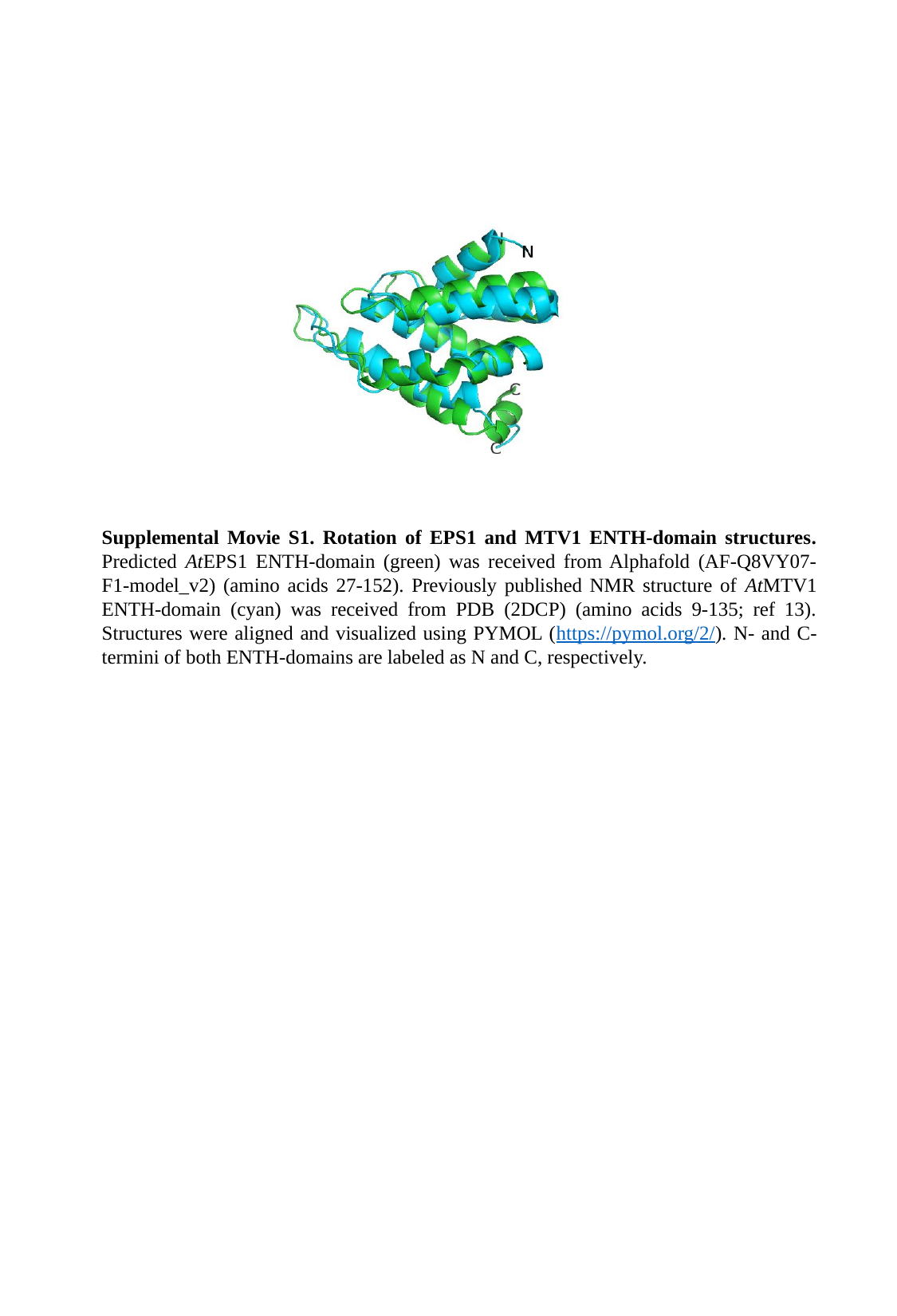

Supplemental Movie S1. Rotation of EPS1 and MTV1 ENTH-domain structures. Predicted AtEPS1 ENTH-domain (green) was received from Alphafold (AF-Q8VY07-F1-model_v2) (amino acids 27-152). Previously published NMR structure of AtMTV1 ENTH-domain (cyan) was received from PDB (2DCP) (amino acids 9-135; ref 13). Structures were aligned and visualized using PYMOL (https://pymol.org/2/). N- and C-termini of both ENTH-domains are labeled as N and C, respectively.
